# The role of Poller screws in intramedullary nailing for lower limb extra-isthmic fractures

**DOI:** 10.1186/s13018-026-06900-6

**Published:** 2026-05-07

**Authors:** Zhanyu Yang, Bin Sheng, Delong Liu, Ying Lu, Bin Chen

**Affiliations:** 1 Division of Orthopaedics and Traumatology, Department of Orthopaedics, Nanfang Hospital, Southern Medical University, Guangzhou, 510515 China; 2 Department of Orthopaedics, Hunan Provincial People’s Hospital (the First Affiliated Hospital of Hunan Normal University), No.61 Jiefang West Road, Changsha, Hunan China; 3 Hunan Emergency Center, No. 90 Pingchuan Road, Changsha, Hunan China

**Keywords:** Lower limb fractures, Intramedullary nail, Poller screws, Union, Meta-analysis, Finite element analysis

## Abstract

**Introduction:**

Intramedullary nailing is the preferred treatment for lower limb long bone fractures, but stability is reduced in extra-isthmic fractures due to nail-metaphysis mismatch. Poller screws may help, but their use is controversial. This study evaluates whether Poller screws are associated with improved prognosis in intramedullary nailing for lower limb extra-isthmic fractures and explores their mechanical mechanisms.

**Methods:**

PubMed, EMBASE, Cochrane Library, and Web of Science were searched up to December 2025 using keywords like 'Fracture,' 'Intramedullary nail,' 'Poller screw,' and 'Blocking screw.' Studies were screened, and data were collected for meta-analysis. A 3D tibial model was created, and finite element analysis assessed Poller screws' effect on distal tibial fractures under axial load.

**Results:**

Out of 1134 studies, 5 trials with 413 participants were included. The Poller screw group showed increased union rates (OR = 2.48; 95% CI, 1.13–5.46; *p* = 0.020). Surgery duration increased, but secondary surgical procedures decreased. No differences were found in malalignment, time to union, or infection. Poller screws reduced fracture site displacement by 54.88%.

**Conclusion:**

The use of Poller screws in intramedullary nailing for lower limb extra-isthmic fractures is associated with higher union rates without affecting alignment or healing time. While surgery duration increases, infection remains unchanged, and secondary surgery decreases. The improved healing outcomes may contribute to Poller screws enhancing construct stiffness and reducing micro-motion.

**Supplementary Information:**

The online version contains supplementary material available at 10.1186/s13018-026-06900-6.

## Introduction

A study conducted in the United Kingdom involving 32,900 patients revealed an annual incidence of tibial and/or fibular shaft fractures of 16.9 per 100,000 individuals [1]. Similarly, a nationwide study in Sweden reported an annual incidence of 10 per 100,000 people for femoral shaft fractures [2]. It implies that lower limb long bone fractures remain one of the most common bone injuries and pose a significant clinical challenge. Such injuries can arise from a variety of mechanisms, ranging from low-energy torsional forces during daily activities to high-energy trauma, such as direct impacts from traffic accidents. Treatment strategies are generally categorized into conservative and surgical approaches, depending on factors such as fracture type, location, degree of fragmentation, and extent of soft tissue damage. Surgical fixation is typically recommended for open fractures , severely comminuted fractures, and closed fractures where functional reduction cannot be maintained [3].

Intramedullary nailing is widely regarded as the preferred treatment for extra-articular fractures of long bones, particularly in the lower limb, due to its superior biomechanical stability, facilitation of early weight-bearing, minimal soft tissue and vascular disruption, and positive impact on fracture healing [4–6]. Compared to conventional plating techniques, intramedullary nailing has demonstrated higher union rates and improved functional outcomes [7, 8]. Its indications have expanded to include metaphyseal fractures [9]. Intramedullary nailing not only aligns and stabilizes fractures by filling the medullary canal but also resists bending and rotational forces through the bone-implant interface. However, biomechanical stability may be compromised by discrepancies between nail and metaphyseal diameters, as well as insufficient bone-implant contact, potentially leading to complications such as nonunion and malalignment [10–12]. These complications can result in increased morbidity, additional surgeries, prolonged healing times, social disengagement, and heightened financial burdens [13, 14].

The concept of the "blocking screw" was first introduced by Donald and Seligson et al. [15] to address axial deformities in fractures prone to bending. Kretteck et al. [16] later refined this concept into the "Poller screw," a device that acts as a physical barrier to guide or restrict nail movement. As a convenient, readily available, and minimally invasive surgical tool, the Poller screw narrows the effective diameter of the nail channel, ensuring central placement of the nail within the widened meta-diaphyseal region and providing a tight mechanical fit [17]. Poller screws have evolved from the first-generation technique of simply constructing an intramedullary corridor to the fourth-generation technique of multiplanar placement [18]. Their core mechanism is to functionally reduce the width of the medullary cavity, neutralize shear forces, and increase compressive forces at the fracture site, thereby addressing the insufficient stability caused by the mismatch between the intramedullary nail and metaphyseal diameters [19–21]. Although existing studies have confirmed their potential in reducing nonunion and malunion rates, their clinical application value has not been systematically verified in extra-isthmic fractures, and relevant guidelines still lack uniform standards.

The use of Poller screws has been reported as an effective technique for facilitating fracture reduction, controlling malalignment, and improving the stability of intramedullary nail-bone constructs [22, 23]. Despite its potential benefits, there is a lack of robust data to establish clear guidelines for Poller screw application, leading to reliance on surgeon discretion. To our knowledge, this study is the first to address this issue using evidence-based methods. The primary objective is to evaluate whether Poller screws can improve outcomes in lower limb extra-isthmic fractures treated with intramedullary nailing. A secondary aim is to elucidate the underlying mechanisms through numerical modelling and finite element analysis. Ultimately, this study seeks to establish a standardized framework for the use of Poller screws in clinical practice.

## Methods

### Protocol registration and literature search

This analysis adhered to the guidelines outlined in the Preferred Reporting Items for Systematic Reviews and Meta-Analyses (PRISMA) Statement [24]. Prior to data extraction and analysis, the research protocol was registered with PROSPERO (CRD42023466151). A comprehensive search was conducted across PubMed, EMBASE, the Cochrane Library, and Web of Science from their inception until December 2025. Tailored search strategies were developed for each database, focusing on the following keywords: “Fracture,” “Intramedullary nail,” “Poller screw,” and “Blocking screw” (see Additional file for details). No restrictions were placed on publication date or region, but only studies published in English were included. Reference lists of retrieved articles were also reviewed for additional relevant studies. Duplicates were removed prior to screening.

### Inclusion and exclusion criteria

Studies were included if they met the following criteria: (1) randomized controlled trials (RCTs) or high-quality observational studies; (2) comparisons of intramedullary nailing with and without Poller screws for lower limb extra-isthmic fractures; and (3) reporting of outcomes such as union, nonunion, malalignment, or other relevant indicators. Extra-isthmic fractures were defined based on the criteria used in the included primary studies, specifically referring to fractures of the femur or tibia located in the segment outside the isthmic portion and adjacent to the metaphysis. We acknowledge that specific anatomical landmarks and measurable criteria varied among the included studies, which represents a limitation in reproducibility. Future studies should aim for standardized anatomical definitions and measurable boundaries for extra-isthmic fractures. Exclusion criteria included: (1) studies focusing on other interventions (e.g., revision for nonunion or limb lengthening); (2) studies involving pathologic fractures, deep intramedullary infections, or severe multiple injuries; and (3) studies limited to techniques, patents, biomechanics, finite element analyses, case series, case reports, reviews, surveys, letters, editorials, comments, or conference abstracts. To avoid subject overlap, duplicate studies were identified and excluded based on demographic characteristics, authorship, and institutional affiliations. Titles and abstracts were screened using a non-blinded standardization approach, with final inclusion decisions based on adherence to the inclusion and exclusion criteria. Disagreements were resolved through consensus.

### Research quality assessment

Study quality was evaluated using the Newcastle–Ottawa Scale (NOS), which provides a semi-quantitative assessment based on a star system [25]. All included studies were retrospective cohort studies, as no eligible RCTs were identified. Two independent investigators, blinded to study outcomes, scored each item. The NOS scale ranges from zero to nine stars, with studies scoring fewer than 4 stars deemed low quality and those scoring 5 or more stars considered eligible. The assessment results for each study are summarized in Table 1.

**Table 1:** Characteristics of studies

Study	Publication year	Country	Study type	Quality assessment	Duration	Sample size	Groups	Female (%)	Age (year)	Fracture site	Definition of union and nonunion	Definition of malalignment
Fawdington et al. [30]	2019	UK	Cohort (retrospective)	**** * ***	2 years	30	Poller screws VS no Poller screws	50.0%	39.7(16.3)	Distal Tibia fractures	Union was defined as a Radiological Union Scale for Tibia (RUST) score of 10 or more	Using a joint orientation difference of 2° or more to be indicative of a progressive deformity and a loss of fracture alignment
Guo et al. [31]	2021	China	Cohort (retrospective)	**** * ***	53 months	96	Poller screws VS no Poller screws	29.2%	48.0(18.6)	Proximal or distal fractures of the femur and tibia	Union was defined as the ability to bear full weight without pain, with the callus bridging in 3 of 4 cortices on radiographs while nonunion was defined as the absence of progressive fracture healing for three consecutive months	Angulation > 5°on any plane, rotational deformity > 15°, or shortening > 2 cm
Peat et al. [32]	2021	UK	Cohort (retrospective)	**** * ***	5 years	154	No Poller screws VS 1 Poller screw VS 2 Poller screws	40.3%	41.3(17.7)	Tibia fractures	Nonunion was defined as the absence of clinical and radiological evidence of bone healing at 9 months plus the need for further operative intervention	Not applicable
Schumaier et al. [11]	2019	USA	Cohort (retrospective)	**** ***	6 years	84	Poller screws VS no Poller screws	28.6%	41.7(18.6)	Distal third fractures of the femur	Using radiographic apparent bone gap (RABG) as a nonunion predictor	Not applicable
Song et al. [12]	2019	Korea	Cohort (retrospective)	**** * ***	8 years	49	Poller screws VS no Poller screws	38.8%	39.6(12.1)	Infraisthmal acute femur-shaft fractures	Union was defined as the ability to bear full weight without pain, with callus bridging in 3 of 4 cortices on radiographs	Malunion was defined as more than 5 degrees of angulation on radiographs, more than 15 degrees of malrotation compared to contralateral side range of motion and shortening of more than 2 cm on scanographs
Van Dyke et al. [28]	2018	USA	Cohort (retrospective)	** * ***	46 months	116	Poller screws VS no Poller screws	40.5%	38.6(16.8)	Infraisthmal femur fracture	Union was defined as bridging callus of 3 or more cortices on 2 orthogonal radiographs. Nonunion was defined as the need for any surgical intervention intended to stimulate fracture healings	Not applicable

### Data collection and abstraction

Two investigators independently extracted the following data: title, lead author, publication year, country, study type, duration, sample size, cohorts compared, demographic data (mean age, gender, fracture site), definitions of union and nonunion, and definitions of malalignment. The extracted data are summarized in Table 1. For dichotomous variables, the number of events was recorded, while for continuous variables, the mean and standard deviation (SD) were recorded. When necessary, values were indirectly calculated from *p*-values or confidence intervals. If data from multiple subgroups needed merging, the mean, SD, and sample size of the subgroups were combined.

### Outcome measures

Outcomes reflecting surgical risk and prognosis were defined as follows:

1. Incidence of union and nonunion: Nonunion events included only osseous nonunion during follow-up; delayed union was excluded.
2. Incidence of malalignment: Defined as per Table 1.
3. Time to union (weeks): Time from surgery to radiographic union.
4. Duration of surgery (minutes): Total time for all surgical procedures.
5. Incidence of infection: Included both superficial and deep infections without differentiation.
6. Incidence of secondary surgical procedures: Included revision for nonunion and removal of internal fixation due to pain.

### Meta-analysis methodology

Meta-analyses were performed to calculate odds ratios (ORs) or weighted mean differences (WMDs) with 95% confidence intervals (CIs) using the Mantel–Haenszel statistical method. Studies reporting no events in any group were excluded from OR calculations, as recommended by the Cochrane Handbook. Heterogeneity was assessed using the I^2^ statistic. A random-effects model was applied for significant heterogeneity (*p* < 0.05), while a fixed-effects model was used in its absence. Publication bias was evaluated using funnel plots. Sensitivity analyses were conducted by excluding studies with confounding variables or differing characteristics. All analyses were performed using Review Manager 5.4, with *p* < 0.05 considered statistically significant.

### Model construction and measurement

Tibial computed tomography (CT) data were obtained from a 41-year-old male weighing approximately 65 kg using a Siemens 64-row CT scanner with a slice thickness of 0.7 mm. The CT images, saved in Digital Imaging and Communications in Medicine (DICOM) format, were imported into Mimics 21.0 software (Materialise, Leuven, Belgium) to create a three-dimensional tibial model, which was exported in STL format. These files were processed in Geomagic Wrap 2021 (Geomagic, USA) for smoothing, meshing, noise reduction, and surface fitting. Boolean operations were used to construct three-dimensional models of cortical and cancellous bones. The models were imported into SolidWorks 2023 (Dassault, France) to evaluate the characteristics of the extra-isthmic fracture model. Three-dimensional geometric models of intramedullary nails, with and without Poller screws, were created in SolidWorks 2023. In the three-dimensional model, the screw was placed on the stress side of the fracture line. A 3.5 mm cortical bone screw was selected and kept parallel to the intramedullary nail to form a "tunnel" structure for guiding the central fixation of the intramedullary nail, which conforms to the "epicentric principle" to maximize the compressive force at the fracture site [21]. The assembly of intramedullary nails and fracture models was completed, and the geometric model file was exported. The primary distinction between the two groups was the presence of a Poller screw. Finally, the geometric fracture models and internal fixation structures were imported into ANSYS Workbench 2023 (ANSYS, USA) for computational analysis. A sensitivity analysis was conducted to determine the optimal element size for the model. The ideal mesh size and quality were identified through this analysis. All materials were assumed to be continuous, isotropic, and linearly elastic [26]. The elastic modulus for bones and implants is provided in Table 2. The finite element model was constructed based on a single tibial CT scan and did not include the fibula. Loading conditions were limited to axial compression. To validate the finite element model, an intact tibia model was reconstructed, and a vertical load of 500 N was applied to the tibial plateau, based on data from a published experimental study [27]. The results closely matched those of the earlier study, confirming the model’s accuracy and reliability for future research.

**Table 2:** Properties of the materials

Material	Poisson’s ratio	*E* (MPa)
Cortical bone	0.3	17,000
Cancellous bone	0.3	700
Intramdullary nail and Poller screw	0.34	107,000

### Finite element analysis

In the finite element models, an axial force of 500 N was applied to the medial and lateral compartments of the tibial plateau, with the distal end of the tibia fully constrained. Frictional contact conditions were applied to the interaction surfaces between the nail and screws, the nail and bone, and the fracture surfaces, with friction coefficients set to 0.15, 0.1, and 0.3, respectively. The screw-bone interaction was modeled using a bonded contact condition. Displacements and von Mises stress distributions in the tibia and internal fixation were recorded for each group, with variations in these parameters observed across the groups.

## Results

### Study selection

During the initial search, 1134 records were identified from various databases: 26 from the Cochrane Library, 227 from PubMed, 441 from EMBASE, and 440 from Web of Science. After removing duplicates, 786 articles remained. Screening of titles and abstracts led to the exclusion of 743 studies, leaving 43 studies for full-text review. No additional studies were identified from reference lists. Of these, 37 studies were excluded for the following reasons: 12 lacked a control group, and 9 compared different treatment methods.

**Fig. 1:**
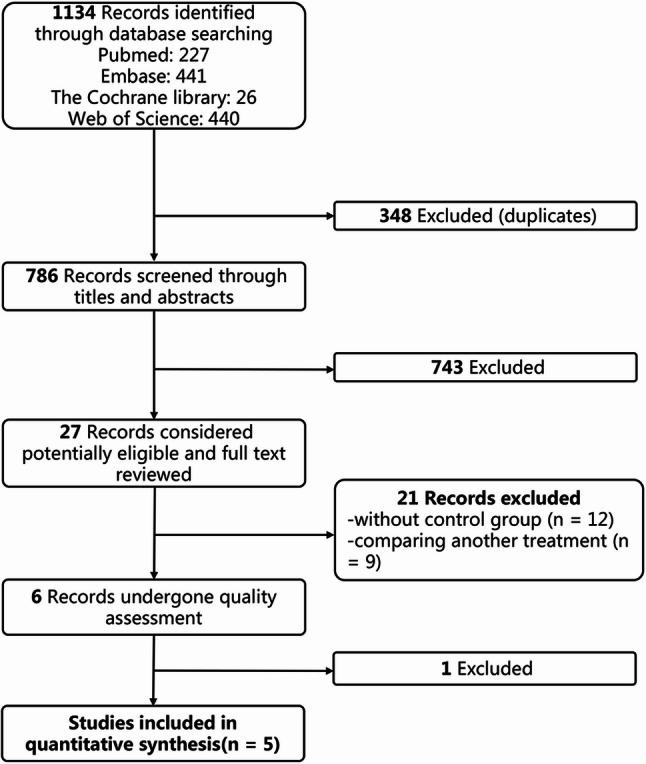
Flowchart illustrating the study selection process

After quality assessment, Van Dyke et al. [28] was assigned 6 stars but exhibited anomalies, including a higher rate of positive results compared to other studies, raising concerns about publication bias. This study also had a significantly higher proportion of type C fractures (OTA/AO classification) [29] in the Poller screw group and a disproportionate number of gunshot wound cases between groups, contributing to excessive heterogeneity. Consequently, this study was excluded from the meta-analysis. Ultimately, 5 studies were included [11, 12, 30–32] (Fig. 1).

### Incidence of union and nonunion

**Fig. 2:**
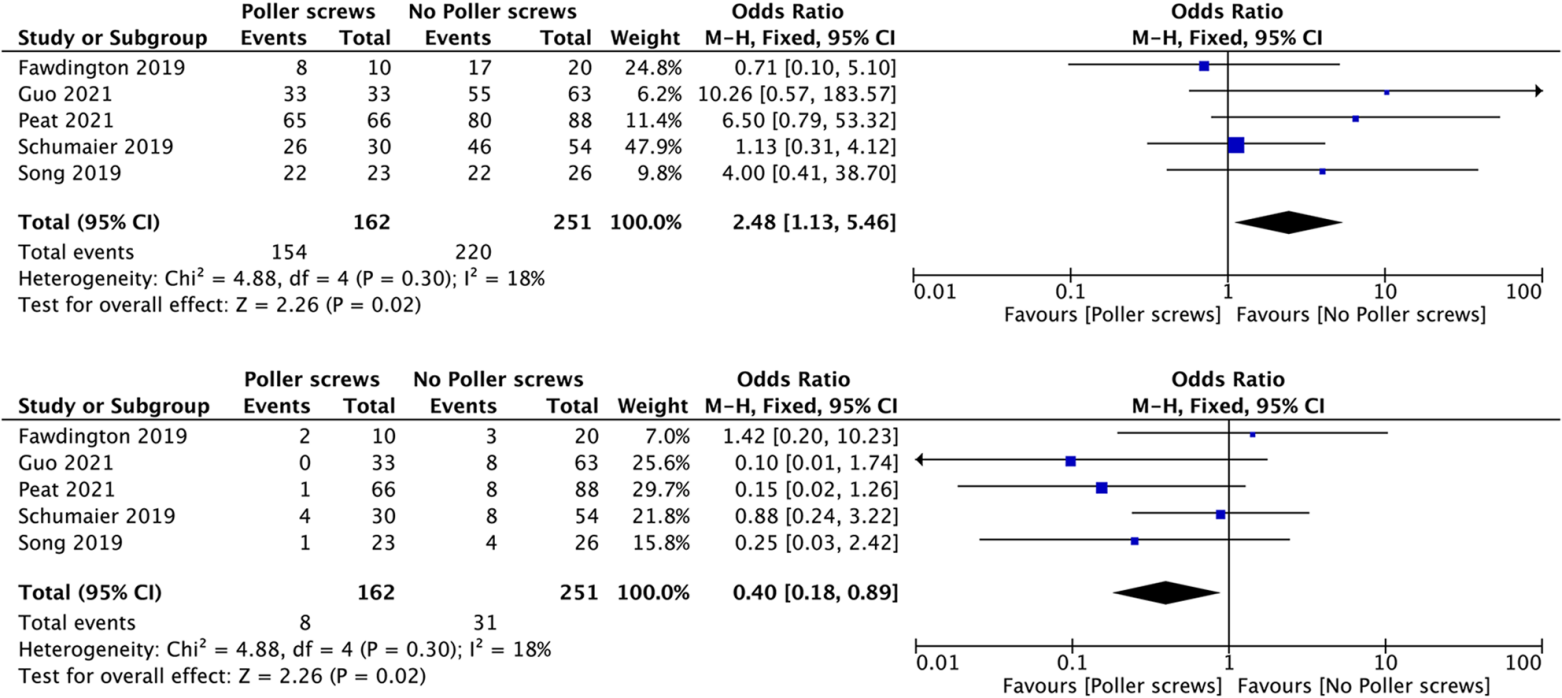
Meta-analysis results for the incidence of union and nonunion

The use of Poller screws significantly increased the occurrence of union (OR = 2.48; 95% CI, 1.13–5.46; *p* = 0.020) and decreased the occurrence of nonunion (OR = 0.40; 95% CI, 0.18–0.89; *p* = 0.020) (Fig. 2). No statistical heterogeneity or publication bias was observed (*p* = 0.30; I^2^ = 18%).

### Incidence of malalignment

**Fig. 3:**
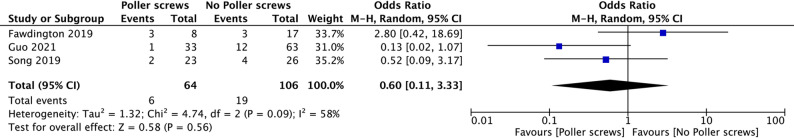
Meta-analysis results for the incidence of malalignment

No significant differences in malalignment were found between the Poller screw group and the control group (OR = 0.60; 95% CI, 0.11–3.33; *p* = 0.560) (Fig. 3). Moderate heterogeneity (*p* = 0.09; I^2^ = 58%) was observed, prompting the use of a random-effects model. Further analysis revealed that Guo et al. [31] and Song et al. [12] applied consistent malalignment criteria, whereas Fawdington et al. [30] utilized distinct criteria. To evaluate the influence of the study by Fawdington et al. [30] on the pooled outcomes, we performed a sensitivity analysis following its exclusion. Sensitivity analysis identified Fawdington et al. [30] as the primary source of heterogeneity. Excluding this study reduced heterogeneity (*p* = 0.32; I^2^ = 0%), but the overall results remained unchanged (OR = 0.29; 95% CI, 0.07–1.14; *p* = 0.08). These findings suggest that despite variability in outcome definitions, the primary conclusion of this study remains robust and unaltered.

### Time to union

**Fig. 4:**
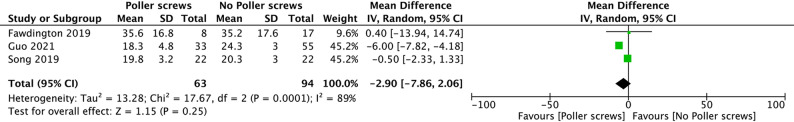
Meta-analysis results for the time to union

No significant differences in time to union were observed between the two groups (WMD =  − 2.90; 95% CI, -7.86–2.06; *p* = 0.25) (Fig. 4). Significant heterogeneity (*p* = 0.0001; I^2^ = 89%) was noted, necessitating a random-effects model. Sensitivity analysis identified Guo et al. [31] as the primary source of heterogeneity. Excluding this study eliminated heterogeneity (p = 0.90; I^2^ = 0%), with no change in overall results (WMD = −0.49; 95% CI, -2.30–1.33; *p* = 0.60).

### Duration of surgery

**Fig. 5:**
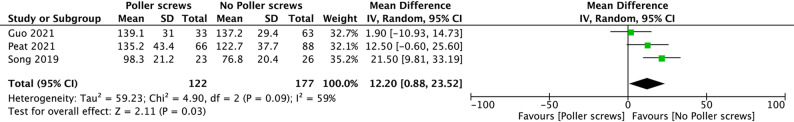
Meta-analysis results for the duration of surgery

The insertion of Poller screws was associated with a significant increase in surgical duration (WMD = 12.20; 95% CI, 0.88–23.52; *p* = 0.03) (Fig. 5). Moderate heterogeneity *p* (= 0.09; I^2^ = 59%) was observed, leading to the use of a random-effects model. Sensitivity analysis excluding Guo et al. [31] reduced heterogeneity (*p* = 0.32; I^2^ = 1%) without altering the results (WMD = 17.51; 95% CI, 8.74–26.27; *p* < 0.0001).

### Incidence of infection

**Fig. 6:**
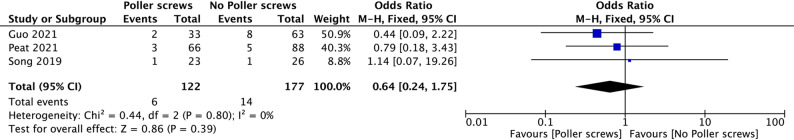
Meta-analysis results for the incidence of infection

No significant differences in infection rates were observed between the two groups (OR = 0.64; 95% CI, 0.24–1.75; *p* = 0.39) (Fig. 6). No statistical heterogeneity (p = 0.80; I^2^ = 0%) or publication bias was detected.

### Incidence of secondary surgical procedures

**Fig. 7:**
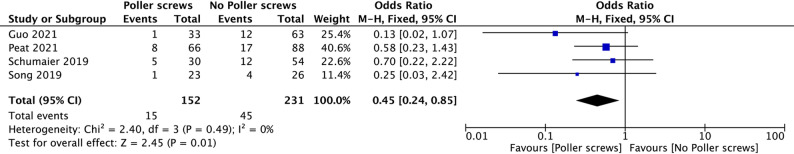
Meta-analysis results for the incidence of secondary surgical procedures

The use of Poller screws significantly reduced the need for secondary surgical procedures (OR = 0.45; 95% CI, 0.24–0.85; *p* = 0.01) (Fig. 7). No statistical heterogeneity (*p* = 0.49; I^2^ = 0%) or publication bias was observed.

### Finite element analysis

#### Model construction and validation

**Fig. 8:**
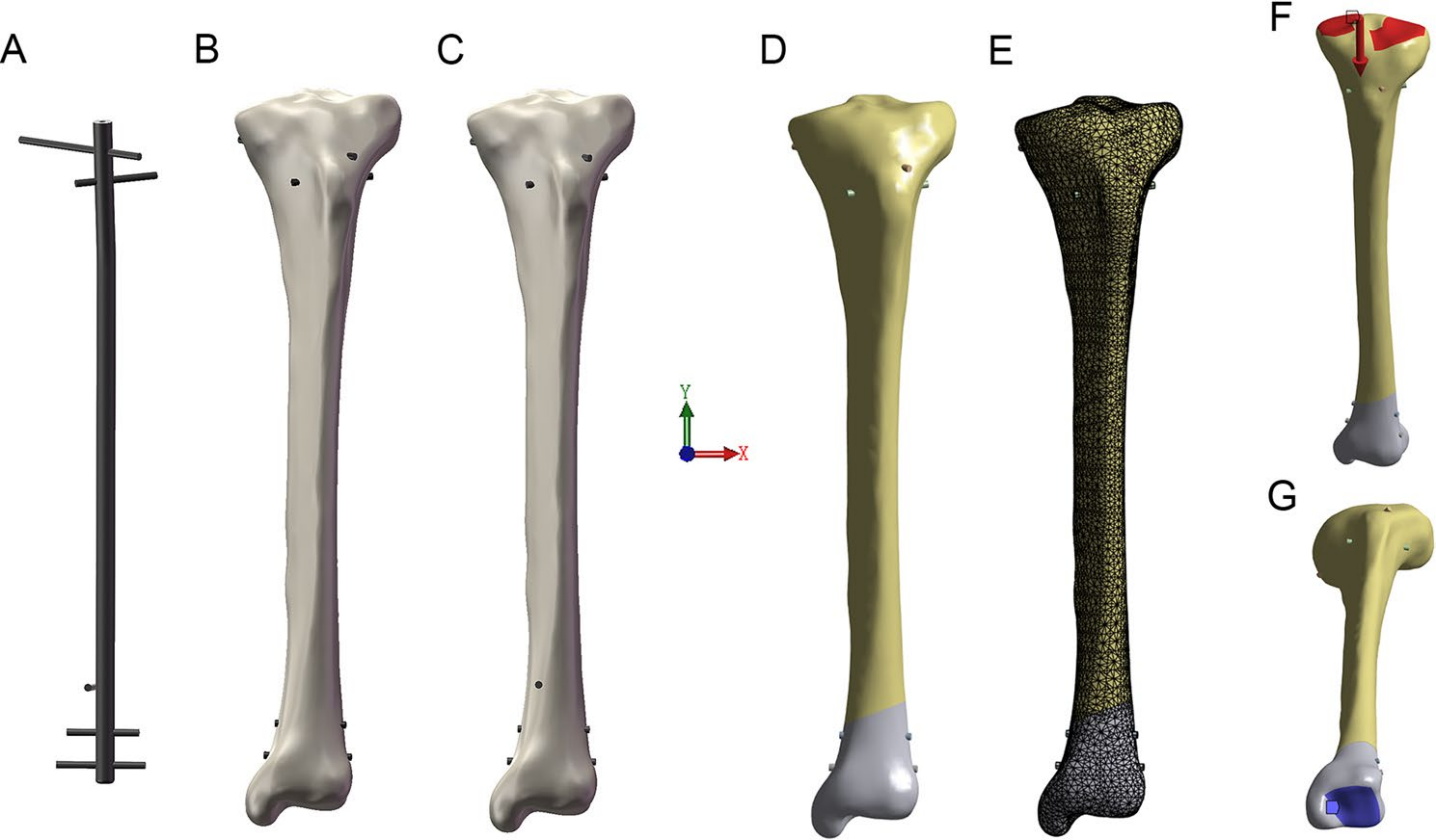
Finite element models and configurations. **A** Intramedullary nail construct with a Poller screw. **B**, **C** Tibia-nail constructs with and without a Poller screw. **D**, **E** Distal tibial fracture model assembled with an intramedullary nail and its meshed construct. **F**, **G** Boundary condition for the application of axial load on tibia condyles and boundary condition for the constrained distal tibia and talus

Finite element models of distal tibial fractures with and without Poller screws were constructed (Fig. 8). A sensitivity analysis determined the optimal element size for the model [33, 34]. Five element sizes (1.0, 1.5, 2.0, 2.5, and 3.0 mm) were tested, with the 1.0–mm mesh selected based on minimal differences in maximum equivalent stress (3.2% difference compared to 1.5–mm mesh). The model without Poller screws consisted of 660,612 nodes and 374,685 elements, while the model with Poller screws contained 665,093 nodes and 376,140 elements.

#### Model displacement

**Fig. 9:**
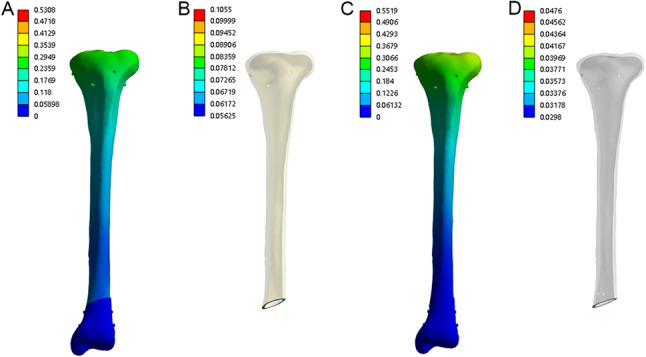
Displacement of two distal tibial fracture models with or without Poller screw. **A**, **B** Total displacement and fracture site displacement of the model without a Poller screw. **C**, **D** Total displacement and fracture site displacement of the model with a Poller screw

Figure 9 illustrates the deformation in the two models. At the fracture site, displacement was 0.1055 mm in the non-Poller screw group and 0.0476 mm in the Poller screw group, representing a 54.88% reduction. Deformation in both groups was concentrated at the locking screws.

#### Stress distribution

**Fig. 10:**
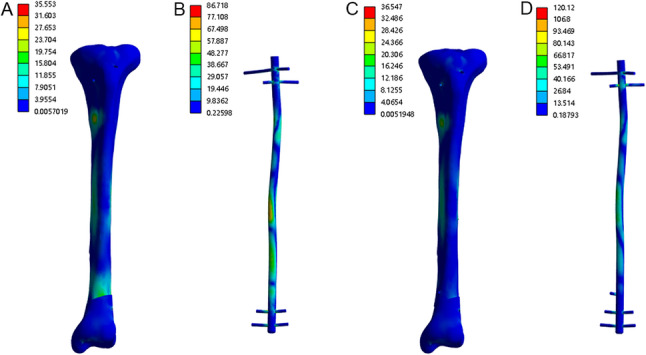
Stress distribution of two distal tibial fracture models with or without Poller screw. **A**, **B** Stress distribution in the tibia and implants for the model without a Poller screw. **C**, **D** Stress distribution in the tibia and implants for the model with a Poller screw

Figure 10 shows the von Mises stress distribution. The maximum stress in implants was 120.12 MPa in the Poller screw group and 86.718 MPa in the non-Poller screw group. For bones, the maximum stress was 36.547 MPa in the Poller screw group and 35.553 MPa in the non-Poller screw group.

## Discussion

The widespread adoption of intramedullary nailing for lower limb long bone fractures underscores its efficacy in treating most fractures. However, studies have highlighted an increased incidence of complications such as varus, valgus, and torsional deformities, delayed union, and nonunion, particularly in fractures involving the extra-isthmic portion [10, 12, 35]. Nonunion rates for femoral shaft fractures treated with intramedullary nailing range from 0.5 to 12.5% [36–41], while for tibial fractures, they can reach 5 to 25% [42–44]. In elderly men with open fractures, nonunion rates can be as high as 41% [45]. Malalignment is also prevalent, with incidences of 14 to 58% in tibial fractures and 10 to 30% in femoral shaft fractures treated with intramedullary nailing [46–48]. These complications often arise in the extra-isthmic region due to the mismatch between the nail diameter and the metaphyseal intramedullary canal, which lacks sufficient stability. Poller screws have emerged as a potential solution by guiding the nail along the correct path and blocking incorrect canal alignment. However, there is no consensus or established guidelines for their use, and significant controversy persists regarding their efficacy in treating long bone extra-isthmic fractures [11, 28, 31].

In this study, 786 records were identified after deduplication. Following the application of predefined inclusion and exclusion criteria and a thorough review of full-text articles, six studies were initially considered. However, the study by Van Dyke et al. [28] was excluded due to significant confounding factors, including a disproportionately high nonunion rate and an overrepresentation of type C fractures and gunshot injuries, which limited its generalizability. Ultimately, five studies comprising 413 participants were included in the meta-analysis.

Our findings demonstrate that the use of Poller screws significantly increases the incidence of union and reduces the incidence of nonunion in patients undergoing intramedullary nailing. These results align with previous studies attributing complications to instability caused by the mismatch between nail and metaphyseal diameters [22]. Poller screws mitigate this mismatch, enhancing stability and making intramedullary nailing more viable for distal tibial fractures [49]. They are also effective in maintaining alignment and stability in proximal tibial fractures [50] and distal femoral shaft fractures when used with retrograde nailing [51]. Similarly, Poller screws improve stability in proximal femoral fractures treated with intramedullary nailing [52]. However, the definitions of union and nonunion varied among the included studies. Notably, significant heterogeneity existed in the definitions of union and nonunion across the included studies, with criteria varying from pure radiographic assessments to combined clinical-radiographic endpoints, and distinct time cutoffs for diagnosing nonunion (Table 1). Specifically, Fawdington et al. [30] adopted a Radiological Union Scale for Tibia (RUST) score ≥ 10 as the radiographic union criterion; Guo et al. [31] and Song et al. [12] defined union as pain-free full weight-bearing combined with radiographic callus bridging in 3 of 4 cortices, with Guo et al. [31] diagnosing nonunion as no progressive fracture healing for 3 consecutive months. Peat et al. [32] defined nonunion as the absence of clinical and radiological healing at 9 months plus the need for reoperation, while Van Dyke et al. [28] used 3 of 4 cortical bridging callus on orthogonal radiographs for union and surgical intervention for impaired healing for nonunion. Schumaier et al. [11] only used radiographic apparent bone gap (RABG) as a nonunion predictor without a clear union definition. This heterogeneity in outcome definitions may have confounded the interpretation of results, representing a significant limitation of the current study.

However, no significant differences were observed in the incidence of malalignment or time to union between patients treated with and without Poller screws. While the Poller screw group showed better outcomes in the forest plot, the results were not statistically significant, likely due to the limited number of studies reporting these outcomes. It should be noted that the malunion analysis in this study has inherent limitations. The > 2° threshold used by Fawdington et al. [30] to define malunion represents a stricter criterion than the conventional > 5° cutoff, potentially leading to an overestimation of risk in the control group. Sensitivity analysis, however, confirmed that this difference in definition did not affect the core conclusions of the study. Future research could benefit from adopting a unified malunion criterion to minimize heterogeneity and enhance comparability across studies. The effectiveness of Poller screws remains debated. Some studies suggest they effectively address malalignment in distal femoral fractures [53] and improve varus/valgus alignment in distal tibial metaphyseal fractures [22]. Others report no significant differences in healing time or malalignment [28], highlighting the need for further research.

The insertion of Poller screws was associated with longer surgical times, attributed to the technical complexity of the procedure [18]. Screw placement must follow the principle of "avoiding the path where the intramedullary nail may stray" [21]. Preoperatively, it is necessary to analyze the direction of fracture forces, place the screw close to the fracture line but avoid comminuted areas, which requires high experience of the surgeon. Repeated attempts at screw placement can increase the risk of complications, emphasizing the need for experienced surgeons at major trauma centers to perform this procedure [21]. While advancements have been made to assist with Poller screw placement, substantial improvements in surgical efficiency remain elusive [6, 54–56]. Future developments may focus on novel devices to facilitate precise and rapid screw insertion.

No differences in postoperative infection rates were observed between the two groups, likely due to the minimal soft tissue dissection required for Poller screw placement. However, repeated screw placement attempts have been linked to increased infection risks in other studies [17, 22, 50]. Notably, the use of Poller screws significantly reduced the incidence of secondary surgical procedures, reflecting improved fracture stability and healing rates, which in turn reduce medical resource consumption and socioeconomic burdens.

Our meta-analysis demonstrates that the use of Poller screws is associated with improved fracture healing rates in extra-isthmic fractures treated with intramedullary nailing. This improvement can be attributed to the ability of Poller screws to address the mismatch between the intramedullary canal and nail diameter, a phenomenon illustrated by a mechanical model [57]. The 'Bell-Clapper Effect' describes this mismatch, where the nail may deviate from the optimal path due to the widened metaphyseal region. Poller screws counteract this effect by increasing the stiffness of the implant-bone construct, reducing interfragmentary motion, and enhancing overall stability [58, 59]. These mechanisms may contribute to improved fracture healing outcomes. To further investigate the underlying mechanisms, we employed finite element analysis. Secondary fracture healing is highly dependent on the initial stability provided by the internal fixation. Both in vitro experiments and computer simulations have demonstrated that micro-motion between fracture fragments acts as a mechanical stimulus that directly influences the healing process [60]. While intramedullary nails generally exhibit higher structural stiffness and less micro-motion compared to plates, as observed in mechanical testing on cadavers [19], this advantage is compromised in lower limb extra-isthmic fractures. The mismatch between the medullary cavity diameter and the intramedullary nail diameter increases micro-motion at the fracture site, which can impede healing [13].

Finite element analysis revealed that the Poller screw significantly enhances axial stiffness by reducing the mismatch between the medullary cavity and nail diameters, thereby improving structural stability. It is important to interpret the finite element analysis findings as supportive rather than definitive. The model simplifies complex biomechanical realities by excluding the fibula, utilizing a single bone geometry, and applying only axial loads. Therefore, these results illustrate a theoretical mechanism of stiffness enhancement but may not fully capture in vivo multi-axial loading conditions that the lower limb experiences during ambulation and weight-bearing. While the addition of Poller screws increases implant stress from 86.718 to 120.12 MPa, this remains well below the stress threshold for titanium materials. At the fracture site, displacement measured 0.1055 mm in the non-Poller screw group and 0.0476 mm in the Poller screw group, corresponding to a reduction of 54.88%. Recent biomechanical evidence indicates that micromotion within 0.15 mm at the fracture site can promote bone healing by providing mechanical stimulation without inhibiting callus formation [61, 62]. Although Poller screws reduced displacement by approximately 55%, the absolute micromotion in both the control (0.1055 mm) and Poller screw groups (0.0476 mm) remained within the range (typically < 0.15 mm) considered favorable for secondary bone healing. Therefore, the clinical benefit of Poller screws should not be attributed solely to the reduction of micromotion. Instead, their primary mechanism likely involves optimizing stress distribution, converting shear forces to compressive forces [18], and preventing the 'bell-clapper' effect, thereby creating a more stable mechanical environment conducive to healing and reducing the risk of nail malposition and subsequent malunion. This mechanism is especially pertinent in mechanically unstable scenarios, such as lower limb extra-isthmic fractures with comminution or large medullary canals, where Poller screws can effectively improve fixation stability and reduce secondary surgery rates. Further studies incorporating clinical follow-up data are warranted to validate the relationship between biomechanical parameters and actual healing outcomes. These findings underscore the Poller screw's role in maintaining proper alignment and stability in intramedullary nailing.

In conclusion, the use of Poller screws appears to be a safe and effective approach for enhancing intramedullary nail performance in lower limb extra-isthmic fractures. By addressing the instability caused by the diameter mismatch, Poller screws are associated with improved fracture healing rates and reduce the need for secondary surgeries. However, their application is not without challenges, prompting ongoing efforts to develop self-limiting intramedullary nails as an alternative treatment.

This study has several limitations. It is crucial to note that all included studies were retrospective cohorts. Consequently, the observed associations should not be interpreted as causal relationships due to the inherent risks of selection bias and confounding by indication. The small number of included trials may introduce publication bias, and inconsistencies in the definitions of union, nonunion, delayed union, and malalignment across studies could influence the results. The methodological quality of the included studies was suboptimal, with only observational studies available, limiting the ability to infer causal relationships. Additionally, the finite element analysis was based on CT scan data from a single sample, excluding the fibula, which may not significantly affect results but introduces potential limitations due to anatomical variations and model quality. Future research should employ multi-phase models to simulate fracture healing more accurately and conduct large-scale, well-designed randomized trials across multiple centers to validate these findings.

## Conclusion

In conclusion, our meta-analysis of five trials involving 413 patients indicates that the use of additional Poller screws in the treatment of lower limb extra-isthmic fractures with intramedullary nailing is significantly associated with improved fracture union rates. This approach does not negatively impact fracture alignment or the time to union. While the insertion of Poller screws may extend surgical duration, it does not increase the risk of infection and significantly reduces the need for secondary surgical procedures. Additionally, finite element analysis provides supportive evidence that Poller screws enhance the stiffness of the bone-implant construct, reduce micro-motion at the fracture site, and mitigate stress shielding, thereby promoting a more favorable healing environment. Due to the retrospective cohort design of all included studies, no causal relationship can be established between Poller screw use and improved fracture healing outcomes. These findings underscore the clinical benefits of Poller screws in improving outcomes for patients with extra-isthmic fractures, although further research is needed to optimize their application and explore alternative solutions.

## Supplementary Information

Below is the link to the electronic supplementary material.

Additional File1.

## Data Availability

The data underlying this article will be shared on reasonable request to the corresponding author.
