# Supplementary material for: The role of Poller screws in intramedullary nailing for lower limb extra-isthmic fractures
Source: J Orthop Surg Res. 2026 May 7;21:397. doi: 10.1186/s13018-026-06900-6 (PMC13361553; doi:10.1186/s13018-026-06900-6)
Supplement: Supplementary file 1 — Additional File1. [file 13018_2026_6900_MOESM1_ESM.docx]

**Additional files**

**1.Pubmed(227)**

#1 fracture[MeSH Terms] OR fracture*[All Fields]

#2 (intramedullary nailing[MeSH Terms]) OR (intramedullary nail*[All Fields])

#3 poller[All Fields] OR block*[All Fields]

#4 #1 AND #2 AND #3

**2.Embase(441)**

#1 ‘fracture’/exp OR fracture*

#2 ‘intramedullary nailing’/exp OR ‘intramedullary nail*’

#3 poller OR block*

#4 #1 AND #2 AND #3

**3.The Cochrane Library(26)**

#1 MeSH descriptor: [Fractures, Bone] explode all trees

#2 (fracture*):ti,ab,kw (Word variations have been searched)

#3 #1 OR #2

#4 MeSH descriptor: [Fractures Fixation, Intramedullary] explode all trees

#5 (intramedullary nail*):ti,ab,kw (Word variations have been searched)

#6 #4 OR #5

#7 (poller):ti,ab,kw (Word variations have been searched)

#8 (block*):ti,ab,kw (Word variations have been searched)

#9 #7 OR #8

#10 #3 AND #6 AND #9

**4.Web of Science(440)**

#1 TS=(fracture*)

#2 TS=(intramedullary nail*)

#3 TS=(poller)

#4 TS=(block*)

#5 #3 OR #4

#6 #1 AND #2 AND #5
